# Butane-1,2,3,4-tetra­carboxylic acid–4,4′-bipyridine (1/2)

**DOI:** 10.1107/S1600536809029237

**Published:** 2009-07-29

**Authors:** Ning Zhang, Xue-Min Shi, Min Shao, Ming-Xing Li

**Affiliations:** a Department of Chemistry, College of Science, Shanghai University, Shanghai 200444, People’s Republic of China; b Instrumental Analysis and Research Center, Shanghai University, Shanghai 200444, People’s Republic of China

## Abstract

The hydro­thermal reaction of butane-1,2,3,4-tetra­carboxylic acid (H_4_butca), 4,4′-bipyridine (bipy) and Mn(SO_4_)_2_·H_2_O afforded a new co-crystal, C_8_H_10_O_8_·2C_10_H_8_N_2_ or H_4_butca·2(bipy), in which strong O—H⋯N hydrogen-bonding and weak π–π stacking [centroid–centroid distance = 3.8459 (19) Å] inter­actions assemble the organic mol­ecules into a three-dimensional supra­molecular framework. C—H⋯O inter­actions are also present. The whole mol­ecule has inversion symmetry.

## Related literature

For the importance of hydrogen-bonding and π-π stacking inter­actions in supra­molecular chemistry, crystal engineering and biological recognition, see: Wang *et al.* (2007 ▶). Many organic co-crystals have been assembled from *N*-heterocycles and polycarboxylic acids, see: Li *et al.* (2007 ▶). For the 1:1 co-crystal H_4_butca·bipy, see: Najafpour *et al.* (2008 ▶). 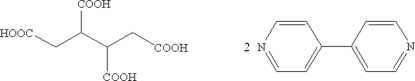


## Experimental

### 

#### Crystal data

C_8_H_10_O_8_·2C_10_H_8_N_2_
*M* *_r_* = 546.53
Triclinic, 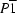

*a* = 7.4435 (11) Å
*b* = 8.6990 (13) Å
*c* = 11.438 (2) Å
α = 99.819 (3)°
β = 105.662 (3)°
γ = 111.361 (2)°
*V* = 633.57 (17) Å^3^
*Z* = 1
Mo *K*α radiation
μ = 0.11 mm^−1^
*T* = 296 K
0.30 × 0.30 × 0.30 mm

#### Data collection

Bruker APEXII CCD area-detector diffractometer
Absorption correction: multi-scan (*SADABS*; Sheldrick, 1996 ▶) *T*_min_ = 0.969, *T*_max_ = 0.969
3287 measured reflections
2196 independent reflections
1721 reflections with *I* > 2σ(*I*)
*R*_int_ = 0.013

#### Refinement

*R*[*F* ^2^ > 2σ(*F* ^2^)] = 0.061
*wR*(*F* ^2^) = 0.176
*S* = 1.07
2196 reflections
183 parameters
H-atom parameters constrained
Δρ_max_ = 0.78 e Å^−3^
Δρ_min_ = −0.30 e Å^−3^

### 

Data collection: *SMART* (Bruker, 2000 ▶); cell refinement: *SAINT* (Bruker, 2000 ▶); data reduction: *SAINT*; program(s) used to solve structure: *SHELXS97* (Sheldrick, 2008 ▶); program(s) used to refine structure: *SHELXL97* (Sheldrick, 2008 ▶); molecular graphics: *SHELXTL* (Sheldrick, 2008 ▶); software used to prepare material for publication: *SHELXTL*.

## Supplementary Material

Crystal structure: contains datablocks global, I, New_Global_Publ_Block. DOI: 10.1107/S1600536809029237/at2847sup1.cif

Structure factors: contains datablocks I. DOI: 10.1107/S1600536809029237/at2847Isup2.hkl

Additional supplementary materials:  crystallographic information; 3D view; checkCIF report

## Figures and Tables

**Table 1:** Hydrogen-bond geometry (Å, °)

*D*—H⋯*A*	*D*—H	H⋯*A*	*D*⋯*A*	*D*—H⋯*A*
C14—H14⋯O4	0.93	2.56	3.476 (3)	168
C9—H9⋯O2	0.93	2.51	3.425 (4)	167
O3—H3*A*⋯N1^i^	0.82	1.78	2.595 (3)	170
O2—H2⋯N2^ii^	0.82	1.84	2.642 (3)	167

Symmetry codes: (i) 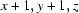; (ii) 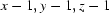.

**Fig. 1.**
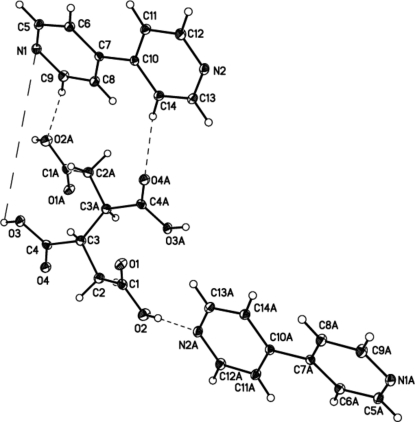
View of (I), showing displacement ellipsoids drawn at the 30% probability level.

**Fig. 2.**
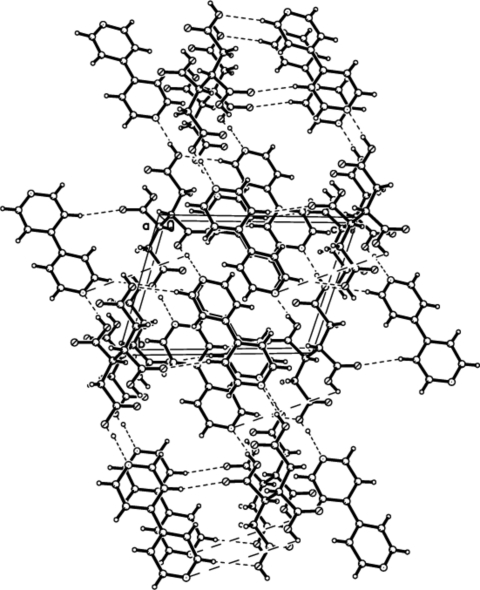
Crystal packing diagram of compound (I)

## supplementary crystallographic information

### Comment

Organic cocrystals involving hydrogen-bonding (Table 1, Fig. 2) and π-π stacking [between the (N1/C5–C9) pyridine rings of 4,4'-bipyridine group, centroid-to-centroid distance = 3.8459 (19) Å] interactions are important in the areas of supramolecular chemistry, crystal engineering, and biological recognition (Wang *et al.*, 2007). Many organic cocrystals have been assembled from N-heterocycle and polycarboxylic acids (Li *et al.*, 2007). 4,4'-Bipyridine (bipy) and butane-1,2,3,4-tetracarboxylic acid (H_4_butca) are important organic ligands in preparing metal complexes.

In our course of preparing ternary complexes containing butane-1,2,3,4-tetracarboxylic acid and 4,4'-bipyridine, a new 1:2 cocrystal compound of H_4_butca.2bipy was prepared unexpectedly. Previously, a 1:1cocrystal of H_4_butca.bipy has been synthesized by solution reaction (Najafpour, *et al.*, 2008). Herein we report the supramolecular framework of the title compound (I) (Fig. 1). The whole molecule has an inversion symmetry which lies on the midpoint of the C3—C3Aa bond of the butane-1,2,3,4-tetracarboxylic acid moiety.

### Experimental

A mixture of H_4_butca (0.0468 g, 0.2 mmol), 4,4'-bipyridine (0.0468 g, 0.30 mmol), Mn(SO_4_)_2_.H_2_O (0.0510 g, 0.3 mmol) and H_2_O (10 ml) was sealed in a 15 ml Teflon-lined stainless-steel reactor, which was heated at 413 K for 72 h. The reaction mixture was cooled to room temperature at a rate of 10 K h^-1^. Light yellow block crystals suitable for X-ray diffraction were obtained in 56% yield (0.0273 g, based on H_4_butca). Analysis calculated for C_28_H_26_N_4_O_8_ (%): C 61.53, H 4.795, N 10.25. Found: C 61.16, H 4.622, N 10.23. IR (KBr pellet, cm^-1^): 3093w, 3040w, 2921w, 1711 s, 1601 s, 1539*m*, 1498*m*, 1414 s,1396 s, 1365*m*, 1285 s, 1217 s, 1128 s, 1071 s, 1005 s, 882*m*, 816 s, 626 s, 534*m*.

### Refinement

All H atoms were located geometrically, with C—H distances of 0.93 - 0.98 Å, O—H distances of 0.82 Å, and allowed to ride on their respective parent atoms with the constraint *U*_iso_(H) = 1.2*U*_eq_(C) and *U*_iso_(H) = 1.5*U*_eq_(O).

### Crystal data

**Table:**

C_8_H_10_O_8_·2C_10_H_8_N_2_	*Z* = 1
*M**_r_* = 546.53	*F*(000) = 286
Triclinic, *P*1	*D*_x_ = 1.432 Mg m^−^^3^
Hall symbol: -P 1	Mo *K*α radiation, λ = 0.71073 Å
*a* = 7.4435 (11) Å	Cell parameters from 1387 reflections
*b* = 8.6990 (13) Å	θ = 2.6–26.6°
*c* = 11.438 (2) Å	µ = 0.11 mm^−^^1^
α = 99.819 (3)°	*T* = 296 K
β = 105.662 (3)°	Block, colourless
γ = 111.361 (2)°	0.30 × 0.30 × 0.30 mm
*V* = 633.57 (17) Å^3^	

### Data collection

**Table:**

Bruker SMART APEXII CCD area-detector diffractometer	2196 independent reflections
Radiation source: fine-focus sealed tube	1721 reflections with *I* > 2σ(*I*)
graphite	*R*_int_ = 0.013
φ and ω scans	θ_max_ = 25.0°, θ_min_ = 2.6°
Absorption correction: multi-scan (*SADABS*; Sheldrick, 1996)	*h* = −8→8
*T*_min_ = 0.969, *T*_max_ = 0.969	*k* = −9→10
3287 measured reflections	*l* = −13→9

### Refinement

**Table:**

Refinement on *F*^2^	Primary atom site location: structure-invariant direct methods
Least-squares matrix: full	Secondary atom site location: difference Fourier map
*R*[*F*^2^ > 2σ(*F*^2^)] = 0.061	Hydrogen site location: inferred from neighbouring sites
*wR*(*F*^2^) = 0.176	H-atom parameters constrained
*S* = 1.07	*w* = 1/[σ^2^(*F*_o_^2^) + (0.0797*P*)^2^ + 0.4629*P*] where *P* = (*F*_o_^2^ + 2*F*_c_^2^)/3
2196 reflections	(Δ/σ)_max_ < 0.001
183 parameters	Δρ_max_ = 0.78 e Å^−^^3^
0 restraints	Δρ_min_ = −0.30 e Å^−^^3^

### Special details

**Table:**

Geometry. All e.s.d.'s (except the e.s.d. in the dihedral angle between two l.s. planes) are estimated using the full covariance matrix. The cell e.s.d.'s are taken into account individually in the estimation of e.s.d.'s in distances, angles and torsion angles; correlations between e.s.d.'s in cell parameters are only used when they are defined by crystal symmetry. An approximate (isotropic) treatment of cell e.s.d.'s is used for estimating e.s.d.'s involving l.s. planes.
Refinement. Refinement of *F*^2^ against ALL reflections. The weighted *R*-factor *wR* and goodness of fit *S* are based on *F*^2^, conventional *R*-factors *R* are based on *F*, with *F* set to zero for negative *F*^2^. The threshold expression of *F*^2^ > σ(*F*^2^) is used only for calculating *R*-factors(gt) *etc*. and is not relevant to the choice of reflections for refinement. *R*-factors based on *F*^2^ are statistically about twice as large as those based on *F*, and *R*- factors based on ALL data will be even larger.

### Fractional atomic coordinates and isotropic or equivalent isotropic displacement parameters (Å^2^)

**Table:**

	*x*	*y*	*z*	*U*_iso_*/*U*_eq_	
C1	−0.1342 (5)	0.6302 (4)	−0.0696 (3)	0.0508 (7)	
C2	−0.0232 (5)	0.7892 (4)	0.0456 (3)	0.0597 (9)	
H2A	−0.1212	0.7939	0.0858	0.072*	
H2B	0.0844	0.7742	0.1058	0.072*	
C3	0.0728 (5)	0.9587 (3)	0.0242 (3)	0.0548 (8)	
H3	0.1316	0.9400	−0.0410	0.066*	
C4	0.2542 (5)	1.0895 (4)	0.1447 (3)	0.0498 (7)	
C5	−0.2914 (4)	0.4885 (4)	0.4266 (3)	0.0479 (7)	
H5	−0.3917	0.4111	0.4485	0.057*	
C6	−0.1373 (4)	0.6347 (4)	0.5207 (3)	0.0449 (7)	
H6	−0.1346	0.6548	0.6038	0.054*	
C7	0.0146 (4)	0.7522 (3)	0.4900 (2)	0.0368 (6)	
C8	−0.0007 (4)	0.7138 (4)	0.3640 (3)	0.0490 (7)	
H8	0.0963	0.7888	0.3387	0.059*	
C9	−0.1600 (5)	0.5644 (4)	0.2767 (3)	0.0543 (8)	
H9	−0.1671	0.5408	0.1928	0.065*	
C10	0.1856 (4)	0.9112 (3)	0.5876 (2)	0.0373 (6)	
C11	0.2302 (4)	0.9316 (4)	0.7163 (3)	0.0467 (7)	
H11	0.1531	0.8442	0.7436	0.056*	
C12	0.3888 (4)	1.0814 (4)	0.8037 (3)	0.0515 (7)	
H12	0.4160	1.0920	0.8897	0.062*	
C13	0.4643 (4)	1.1920 (4)	0.6489 (3)	0.0500 (7)	
H13	0.5455	1.2815	0.6249	0.060*	
C14	0.3093 (4)	1.0477 (4)	0.5543 (3)	0.0452 (7)	
H14	0.2869	1.0409	0.4692	0.054*	
N1	−0.3041 (4)	0.4522 (3)	0.3059 (2)	0.0486 (6)	
N2	0.5053 (3)	1.2116 (3)	0.7720 (2)	0.0480 (6)	
O1	−0.1175 (4)	0.6219 (3)	−0.1724 (2)	0.0646 (6)	
O2	−0.2488 (4)	0.5023 (3)	−0.0413 (2)	0.0675 (7)	
H2	−0.3115	0.4168	−0.1043	0.101*	
O3	0.4001 (3)	1.1949 (3)	0.11635 (17)	0.0535 (6)	
H3A	0.4895	1.2695	0.1816	0.080*	
O4	0.2601 (3)	1.0897 (3)	0.25184 (19)	0.0617 (6)	

### Atomic displacement parameters (Å^2^)

**Table:**

	*U*^11^	*U*^22^	*U*^33^	*U*^12^	*U*^13^	*U*^23^
C1	0.0491 (16)	0.0413 (16)	0.0448 (17)	0.0165 (13)	0.0013 (13)	0.0039 (13)
C2	0.0589 (19)	0.0442 (17)	0.0560 (19)	0.0128 (15)	0.0062 (15)	0.0121 (14)
C3	0.0543 (17)	0.0355 (15)	0.0479 (17)	0.0079 (13)	−0.0013 (14)	0.0065 (12)
C4	0.0508 (17)	0.0385 (15)	0.0391 (16)	0.0143 (13)	−0.0039 (13)	0.0051 (12)
C5	0.0422 (15)	0.0415 (15)	0.0449 (16)	0.0070 (13)	0.0106 (12)	0.0105 (12)
C6	0.0477 (15)	0.0451 (15)	0.0353 (14)	0.0143 (13)	0.0142 (12)	0.0103 (12)
C7	0.0358 (13)	0.0365 (13)	0.0354 (13)	0.0156 (11)	0.0095 (11)	0.0098 (11)
C8	0.0465 (16)	0.0501 (16)	0.0375 (15)	0.0092 (13)	0.0148 (12)	0.0096 (12)
C9	0.0551 (17)	0.0537 (17)	0.0353 (15)	0.0106 (14)	0.0126 (13)	0.0038 (13)
C10	0.0348 (13)	0.0363 (13)	0.0370 (14)	0.0146 (11)	0.0102 (11)	0.0079 (11)
C11	0.0471 (15)	0.0432 (15)	0.0377 (14)	0.0086 (13)	0.0138 (12)	0.0100 (12)
C12	0.0498 (16)	0.0526 (17)	0.0360 (15)	0.0120 (14)	0.0100 (13)	0.0068 (13)
C13	0.0447 (16)	0.0410 (15)	0.0536 (17)	0.0087 (13)	0.0148 (13)	0.0159 (13)
C14	0.0453 (15)	0.0460 (15)	0.0395 (15)	0.0142 (13)	0.0145 (12)	0.0150 (12)
N1	0.0450 (13)	0.0419 (13)	0.0417 (14)	0.0105 (11)	0.0060 (10)	0.0056 (10)
N2	0.0398 (12)	0.0424 (13)	0.0469 (14)	0.0088 (10)	0.0098 (10)	0.0079 (10)
O1	0.0678 (15)	0.0481 (13)	0.0711 (16)	0.0150 (11)	0.0286 (12)	0.0185 (11)
O2	0.0788 (16)	0.0500 (13)	0.0436 (12)	0.0084 (12)	0.0114 (11)	0.0034 (10)
O3	0.0456 (11)	0.0456 (12)	0.0373 (11)	0.0028 (9)	−0.0005 (9)	−0.0005 (9)
O4	0.0599 (13)	0.0513 (13)	0.0424 (12)	0.0047 (10)	0.0019 (10)	0.0097 (9)

### Geometric parameters (Å, °)

**Table:**

C1—O1	1.209 (4)	C7—C10	1.484 (4)
C1—O2	1.285 (4)	C8—C9	1.374 (4)
C1—C2	1.513 (4)	C8—H8	0.9300
C2—C3	1.482 (4)	C9—N1	1.325 (4)
C2—H2A	0.9700	C9—H9	0.9300
C2—H2B	0.9700	C10—C11	1.383 (4)
C3—C4	1.536 (4)	C10—C14	1.395 (4)
C3—C3^i^	1.542 (6)	C11—C12	1.374 (4)
C3—H3	0.9800	C11—H11	0.9300
C4—O4	1.214 (3)	C12—N2	1.327 (4)
C4—O3	1.301 (4)	C12—H12	0.9300
C5—N1	1.332 (4)	C13—N2	1.324 (4)
C5—C6	1.377 (4)	C13—C14	1.373 (4)
C5—H5	0.9300	C13—H13	0.9300
C6—C7	1.393 (4)	C14—H14	0.9300
C6—H6	0.9300	O2—H2	0.8200
C7—C8	1.386 (4)	O3—H3A	0.8200
			
O1—C1—O2	124.5 (3)	C6—C7—C10	121.7 (2)
O1—C1—C2	126.0 (3)	C9—C8—C7	119.6 (3)
O2—C1—C2	109.5 (3)	C9—C8—H8	120.2
C3—C2—C1	117.3 (3)	C7—C8—H8	120.2
C3—C2—H2A	108.0	N1—C9—C8	123.5 (3)
C1—C2—H2A	108.0	N1—C9—H9	118.3
C3—C2—H2B	108.0	C8—C9—H9	118.3
C1—C2—H2B	108.0	C11—C10—C14	116.6 (2)
H2A—C2—H2B	107.2	C11—C10—C7	121.5 (2)
C2—C3—C4	110.8 (2)	C14—C10—C7	121.8 (2)
C2—C3—C3^i^	116.5 (3)	C12—C11—C10	119.8 (3)
C4—C3—C3^i^	108.9 (3)	C12—C11—H11	120.1
C2—C3—H3	106.7	C10—C11—H11	120.1
C4—C3—H3	106.7	N2—C12—C11	123.5 (3)
C3^i^—C3—H3	106.7	N2—C12—H12	118.2
O4—C4—O3	125.2 (2)	C11—C12—H12	118.2
O4—C4—C3	123.6 (3)	N2—C13—C14	124.0 (3)
O3—C4—C3	111.2 (3)	N2—C13—H13	118.0
N1—C5—C6	123.4 (3)	C14—C13—H13	118.0
N1—C5—H5	118.3	C13—C14—C10	119.2 (3)
C6—C5—H5	118.3	C13—C14—H14	120.4
C5—C6—C7	119.2 (2)	C10—C14—H14	120.4
C5—C6—H6	120.4	C9—N1—C5	117.3 (2)
C7—C6—H6	120.4	C13—N2—C12	116.9 (2)
C8—C7—C6	117.0 (2)	C1—O2—H2	109.5
C8—C7—C10	121.3 (2)	C4—O3—H3A	109.5

Symmetry codes: (i) −*x*, −*y*+2, −*z*.

### Hydrogen-bond geometry (Å, °)

**Table:**

*D*—H···*A*	*D*—H	H···*A*	*D*···*A*	*D*—H···*A*
C14—H14···O4	0.93	2.56	3.476 (3)	168
C9—H9···O2	0.93	2.51	3.425 (4)	167
O3—H3A···N1^ii^	0.82	1.78	2.595 (3)	170
O2—H2···N2^iii^	0.82	1.84	2.642 (3)	167

Symmetry codes: (ii) *x*+1, *y*+1, *z*; (iii) *x*−1, *y*−1, *z*−1.
